# The Path from Survey Development to Knowledge Activism: A Case Study of the Use of a Physical Loads Survey in a Retail Workplace

**DOI:** 10.1177/10482911221074680

**Published:** 2022-02-04

**Authors:** Nicolette Carlan, Terri Szymanski, Jennifer Van Zetten, Margo Hilbrecht, Philip Bigelow

**Affiliations:** 1 153482 University of Waterloo Faculty of Applied Health Sciences, Waterloo, Ontario, Canada; 2 415316 Laurentian University Faculty of Arts, Sudbury, Ontario, Canada; 3 113665 Ontario Public Service Employees Union, North York, Ontario, Canada

**Keywords:** union participation, MSDs, knowledge creation

## Abstract

Workers at a multi-site retailer were concerned that they were experiencing higher than anticipated work-related musculoskeletal disabilities (MSDs). They approached union leadership and academic researchers and a Participatory Action Research (PAR) project was developed which culminated in a targeted online Physical Loads Survey (PLS). The goal was to initiate discussions to design a preventative collaborative ergonomic program. Survey results confirmed that during a shift, workers had significant exposure to standing, carrying loads of more than 25 lbs, pushing and pulling loads greater than 225 lbs, and repetitive arm and hand movements. The successful survey was the first step in the development of a proactive health and safety program. The union proceeded without management participation and was able to move beyond knowledge creation to knowledge activism and change.

## Introduction

Since its creation in 2004, the Centre of Research Expertise for the Prevention of Musculoskeletal Disorders (CRE-MSD), based at the University of Waterloo, has developed collaborative research relationships with employers, union groups, and other advocacy organizations. Together with workplace parties, CRE-MSD conducts research to limit exposures to physical loads and potentially prevent musculoskeletal disabilities (MSDs).

One project was the development of a Physical Loads Survey (PLS), which was initially modeled on Dr. Barbara Silverstein's work in Washington State. Silverstein created a tool to assess the prevalence and magnitude of exposures to physical loads using the entire workplace as the unit of observation. The PLS identified and documented the most common physical loads, which could lead to work-related (MSDs). including force, awkward posture, repetition, vibration, and temperature.^1^ By engaging knowledgeable workplace parties to complete the survey, the findings were representative of exposures of employees across the state. While the survey was being developed, a statewide ergonomic rule was introduced, which included an extensive phase-in period with demonstration projects and the development of training material. During and after the phase-in of the PLS, the researchers found that exposure to physical loads and related injuries was reduced. When the ergonomic rule was subsequently repealed those improvements did not continue.^2^ The research demonstrated not only the potential impact of a statewide approach to MSD prevention, but also the utility of a population-based survey of workplace physical loads.

Following that model, CRE-MSD's research team, which included a Health and Safety Officer (HSO) from one of the largest unions in Ontario, created a validated tool for benchmarking physical loads and refined the tool to be easily used by workplace parties in Ontario. The modified survey allowed workers or managers to document the exposure to physical loads in an individual workplace. Findings from the original Ontario study indicated good to moderate agreement between the findings of the workers or managers, who were Joint Health and Safety Committee (JHSC) representatives, and the team ergonomist That research team concluded that valid results could be achieved by having work-site Health and Safety Representatives (HSRs) complete the PLS, with or without professional consultations.^3^ The advantages of this approach are twofold. First, HSRs can use the survey to supplement worker-driven MSD-related prevention recommendations to the employer. Second, the survey increases HSRs’ role in the decision-making process, which in turn increases the probability that workers will accept new ergonomic interventions.^4–7^

## Background for This Study

The Ontario Public Service Employees Union (OPSEU) represents approximately 180,000 members in a variety of occupations including health care, education, emergency services, social services, faculty and support staff in colleges, and workers in the government-operated liquor retailer. The Liquor Board Employees Division (LBED) is a substantial component of OPSEU with approximately 8,000 members. There is significant exposure to physical loads in more than 500 retail locations and multiple warehouses represented by the union. For example, according to union records, in 1 warehouse, the staff moves (in and out) 2.4 million bottles of liquor (wine and spirits) monthly. Most of the LBED membership are retail workers, who have a variety of duties including stocking shelves, operating cash, and assisting customers. Warehouse employees are responsible for shipping and receiving bulk orders. This LBED workforce accounts for approximately 4% of the union's membership but accounts for approximately 25% of the union's workers’ compensation appeals, the majority of which are for MSDs.

The health and safety infrastructure in this workplace is complex because it includes both statutory and contractually negotiated requirements. In this jurisdiction, the Ontario Occupational Health and Safety Act (OHSA) requires any workplace with 20 or more employees to have a JHSC which keeps records and meets quarterly. Legislated membership on the JHSC includes management and union-selected committee members. The employer must provide certification training to (at least) 1 employer and 1 worker member of a JHSC. Workplaces with fewer than 20 but more than 5 individuals regularly employed must have a worker health and safety representative selected by the union (if the workers are unionized), but the representative is not entitled to receive certification training. OPSEU also selects HSR in workplaces with 5 or fewer employees, even though there is no legislated mandate for these representatives and no right to receive certification training. Few locations, among the approximately 500 work sites, in this organization are large enough to have JHSCs. Most of those JHSCs are made up of only 1 worker member and 1 employer member because most sites have fewer than 50 people employed. Most sites have 1 worker HSR only with no requirement for a designated employer representative. The HSR conducts the legally mandated site-specific activities but is not entitled to meetings, minutes, or certification training. Other sites have HSRs with no legal mandate, so they submit official recommendations and assert rights for other OHSA activities subject to the goodwill and agreement of the employer. Therefore, the health and safety structures (and training requirements) within the organization vary greatly and operate independently and site by site in accordance with health and safety legislation and labor agreement.

The union has also negotiated a Provincial Health and Safety Committee (PHSC) which addresses issues that occur in multiple work sites and attempts to streamline communications among the independent HSRs operating in work sites of the same organization. The PHSC also acts as a central forum to consider provincial issues and disputes forwarded by the sites, but it is not considered by the employer to be a JHSC with rights or activities mandated by OHSA (so its recommendations are not considered official).

HSRs from across the province attended a presentation on the PLS tool by CRE-MSD at the 2015 Repetitive Strain Injury Awareness Day in Toronto and envisioned the application of the tool in their workplaces. They approached OPSEU's HSO to contact CRE-MSD and to help pilot the PLS. The HSRs wanted to document workplace hazards and create technical knowledge that would be the basis for ergonomic improvements. The local representatives, together with provincial union staff, prepared a successful application to secure union funding of approximately $40,000 to facilitate meetings and payment for lost time while local representatives were participating in the project.

Previously, the employer had initiated ergonomic prevention programs for this workforce. However, neither HSOs nor local HSRs were consulted prior to the commencement of the programs and had little formal or informal input into measures and procedures to prevent MSDs. Rather, the employer provided updates to the PHSC about pilot projects and initiatives once underway and then collected worker feedback. For example, the union membership was initially encouraged when the employer retained an ergonomist for advice, but that enthusiasm decreased when they learned that the ergonomist's priority was to assist in speedy return-to-work programs and he could only work on prevention in a limited way. In another example, management introduced a new scanner for cashiers which was not tested. During an interview, a worker reported: “You only had to use the scanner for one day and you would know it was no good.” It turned out that the scanner had significant quality problems and was quickly abandoned. Perhaps, it was because of the lack of consultation that these initiatives were not met with enthusiasm.

Against this background, the union membership could have decided to continue to attempt to negotiate management's participation to begin a new proactive provincial MSD initiative. However, because of the history of non-collaboration, the union decided to independently proceed with the survey and did not ask the employer to participate. The union representatives did inform the employer in writing about the survey and offered to share the results upon completion. The union advised the employer that the survey would be emailed to workers at home. The union representatives wanted to make it clear that no company time or equipment would be used to complete the survey. The representatives were concerned that if the members violated any employer rules about at-work activities there might be discipline or retaliation (such as a reduction in hours).

The broad goal of this case study was to create and employ knowledge to improve workplace health and safety. Many researchers including Hall and Abama^8,9^ have identified different forms of knowledge and we have focussed on 3 forms which include:

*Technical or instrumental knowledge* which is based on the knowledge developed by workers doing the job or on the specific findings of researchers. This type of knowledge can be used to solve a specific problem like reducing weights to minimize lifting requirements.
*Strategic or tactical knowledge* goes beyond technical knowledge and addresses process issues. It is aimed at organizational issues which impact the change in processes.
*Political or conceptual knowledge* is the knowledge that creates the possibility of looking at issues from a broader vantage point. It can be used to introduce a collaborative approach to health and safety and prevention activities like prevention through design or to encourage a greater role for the government in health and safety.

The specific goal of this research was to develop and use technical indigenous (workers’) knowledge about the workplace to develop a program to reduce ergonomic hazards. We hoped to establish that a collaborative research team could document ergonomic hazards, which could be the basis for modification of work practices and collateral hazard reduction. The auxiliary goal was to allow HSRs to expand the role of workers beyond technical representation that limited their activities to the tasks defined in the OHSA. We wanted to provide technical knowledge creators (frontline staff HSRs) with the resources to strategically use their newly acquired knowledge to address issues that recur throughout the entire system. Finally, we wanted to set the stage to use the new knowledge politically to develop initiatives across the system to mobilize workers, to persuade and convince managers to address systemic issues, to develop preventative programs, and to gain support from government inspectors.^8,9^

## Methods

The union's interest in the survey was piqued because members were motivated after participating in CRE-MSD's presentation on its PLS. Members opined that their ergonomic hazards were not adequately addressed and saw an opportunity to do something about it. Unfortunately, statistical data concerning MSDs is not readily available in this workplace for several reasons. Although the employer is required to share information about work-related injuries with the union, this reporting is not necessarily uniform. Each of the 500 work sites reports independently and some workplaces do not always share this information with the provincial union. There are also disagreements between the union and the employer about the significance of a relationship to work. If the employer initially determines the disability is not work-related, there is no obligation to share the information about the disability with the union. Notwithstanding the limited statistical data, the union has received multiple reports from its membership from across the province about significant numbers of MSD complaints. There are reports to the union that there are some workplaces where its entire workforce is restricted because of MSDs. Furthermore, the number of LBED MSD appeals to the Workplace Safety and Insurance Board (WSIB) proportionally dwarf similar appeals in other sectors of the union. In addition to this information, there are reports from individual workers, one of whom reported: “When you work here it was only a matter of time before you get injured”. To address the MSD concerns, a mixed research team was created, made up of provincial union HSOs, provincial and local Health and Safety (H&S) representatives, researchers from the university, and colleagues with technical skills to revise and distribute the PLS.

The methodological foundation of this research is Participatory Action Research (PAR). PAR is a methodology that includes the community as well as academic researchers to participate in setting the agenda, designing research, collecting and analyzing the data, and disseminating the results to encourage change. The goal is to create knowledge and provide direct and immediate benefit to the community.^10,11^ A companion process to PAR, Participatory Ergonomics (PE), was also employed in the development of the original and revised PLS. The PE approach recognizes workers are often the stakeholders most familiar with the work processes and the most suited to identify a comprehensive list of workplace hazards. Not only is PE valuable for the indigenous knowledge it brings to a project, but it also provides workers with the power and influence to create change.^12–14^ This research plan consisted of 3 components: independent research about the workers’ compensation system and the ergonomic hazards in this workplace, union and worker reports, and survey results. The University of Waterloo Office of Research reviewed the research and granted ethics clearance (ORE #21355).

The project began with several meetings with a designated research team. The project team worked on the PLS and dissemination plans. With financial support from the union, the research team held 3 meetings across the province with 56 HSRs from individual work sites to introduce the project. Notably, some of the HSRs had not previously met as groups, so it was an opportunity for them to express concerns and network. At each meeting, the CRE-MSD researcher introduced the purpose of the survey, provided instructions on how to complete the survey, did a demonstration of the survey, and answered questions. During those meetings, the HSRs provided insight into working conditions, which were useful in the analysis.

Relying on the previously validated surveys, the PLS was designed to identify 26 types of loads, eg, carry loads, trunk flexed, lift > 23 kg, lift people, hand above the shoulder, repetitive arm, power grasp, computer use, standing, driving off-road, kneel/squat, and high vibration. The original PLS was structured to estimate the exposure to hazards for the entire workplace. With significant input from the project committee, the PLS was modified to allow workers to record their exposure and did not require them to estimate the exposure for the entire workplace. The original survey also assumed a standard work schedule, and the frequency/duration of exposure was measured in frequency/shift. After the preliminary 100 responses, we learned that hours of work were not standard for the majority of LBED members. This caused confusion because some shifts were only 2 hours long and that complicated the completion of the survey. We changed the measurement of exposure to hazard to include times/shift and/or an estimate of frequency—often, occasionally, and never. After the survey was modified, it was emailed to all identified LBED members. We did not track the number of original emails that were sent. The final PLS is provided as an Online Supplemental Material available on the *New Solutions* website (SAGE Publication).

After the survey, we developed a selective sample of 10 participants representing different groups (eg, management, casual, warehouse, and full-time employees) and conducted interviews. The interviews were conducted by phone, with each interview taking approximately 23 min. They were recorded and the interviewers took detailed notes. The interviews included questions about the survey itself, such as ease of completion, the most common causes of injury, the processes in the workplace that were potentially hazardous and could result in workplace injuries, illnesses, or fatalities, and barriers to improving health and safety.

We received 428 completed surveys. We used REDCap electronic data capture tools hosted at the University of Waterloo to collect and manage the data.^15,16^ This server is protected by the university's firewall and data is protected through a strict security and access control policy. Only authorized researchers with Research Ethics Approval were able to view the data. Authorized REDCap researchers monitored the input and provided an analysis of the data.

After the results of all the data had been analyzed, the university researcher prepared a brief written report addressing the issues of importance and it was presented to the PHSC. At that meeting, the university researcher also presented a report from a similar workplace, which had introduced a proactive ergonomic process.^17^ No joint labor and management actions have taken place since that PHSC meeting. The project team continues to pursue the issue. The item “University of Waterloo Ergonomic Study” appears in provincial committee minutes for 9 quarterly meetings from February 9, 2017 to February 20, 2020.

## Findings

Comments from union members at the meetings and the results of the qualitative interviews provided significant information in addition to quantitative survey results. The qualitative data revealed that in this organization, several factors warrant consideration when designing a proactive MSD program: the complicated health and safety infrastructure, the workers’ compensation system, the physical locations of and organization of the work sites, and the full or part-time status of the employees.

### The Survey Results

The PLS was distributed to all LBED locals to contact their members and through mass union emails. We received responses from 428 employees. There was a mix of employees from different job classifications: 71 casual, 37 managers, 13 warehouse, and 307 retail workers. We did not attempt to contact fixed-term employees.

Part-time staff, which comprise approximately 70% of the workforce, represents only 16% of the responses. One explanation offered by the HSRs was that part-timers were concerned about their relationship with management and reluctant to respond. According to part-time staff who sat on the project team, part-time staff rely on and value the goodwill of management to be assigned shifts each week. The study participants explained that part-time workers feared that if management knew that they were engaged in a union activity, it might make them stand out (or identify them as activists), which could result in retaliation, but not discipline, which could potentially reduce the number of shifts that they would be offered in future.

**Figure 1.**
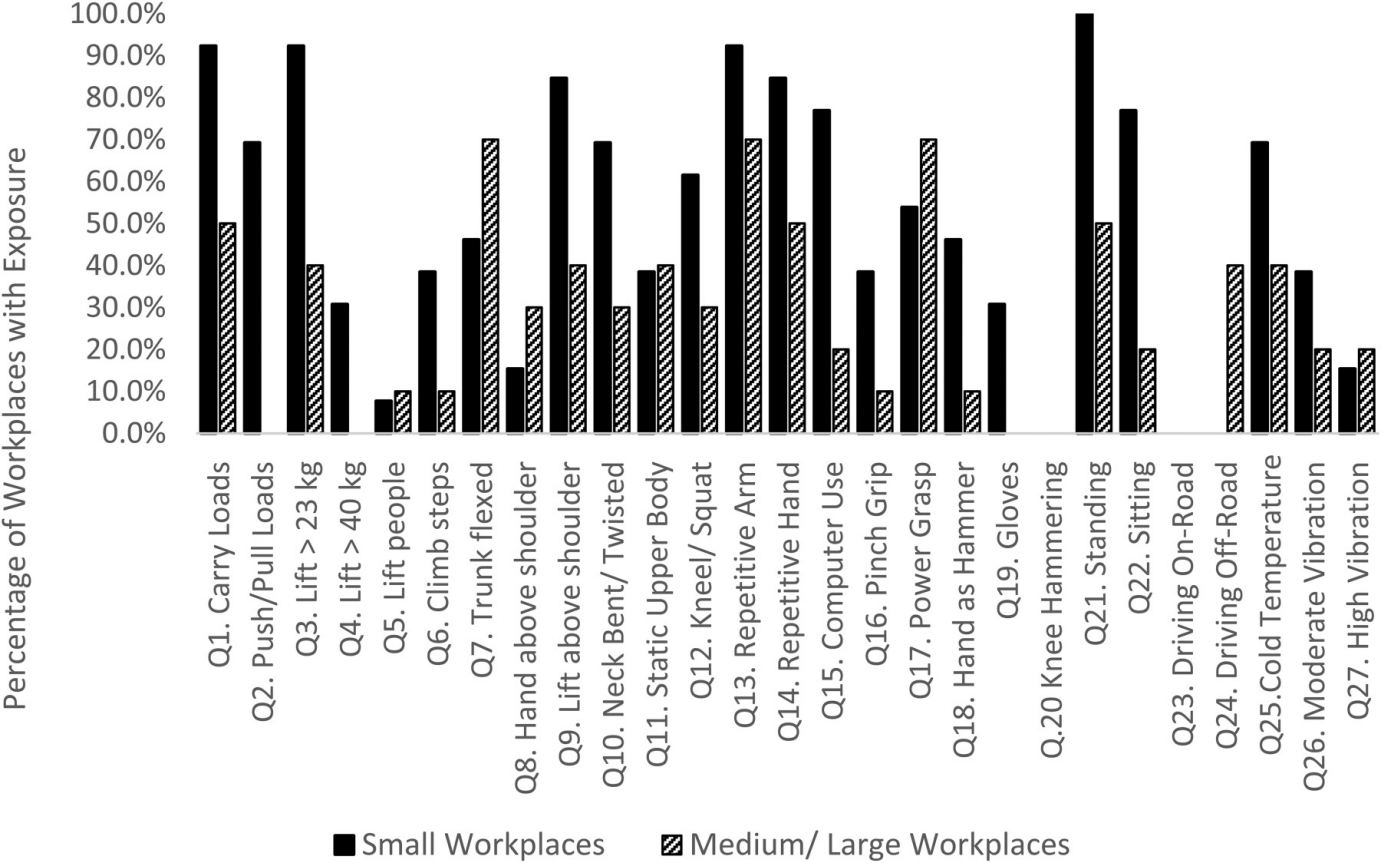
Comparison of physical load exposure between small and medium/large workplaces.

Figure 1 shows the exposures to physical loads according to the size of the workplace. Workers in small workplaces were required to carry heavy loads 90% of the time compared to workers in medium and large workplaces who carried heavy loads only 50% of the time. The same kind of difference is noted when workers in small workplaces reported standing approximately 90% of the time compared to workers in medium and large workplaces who reported standing slightly less than 50% of the time.

**Figure 2.**
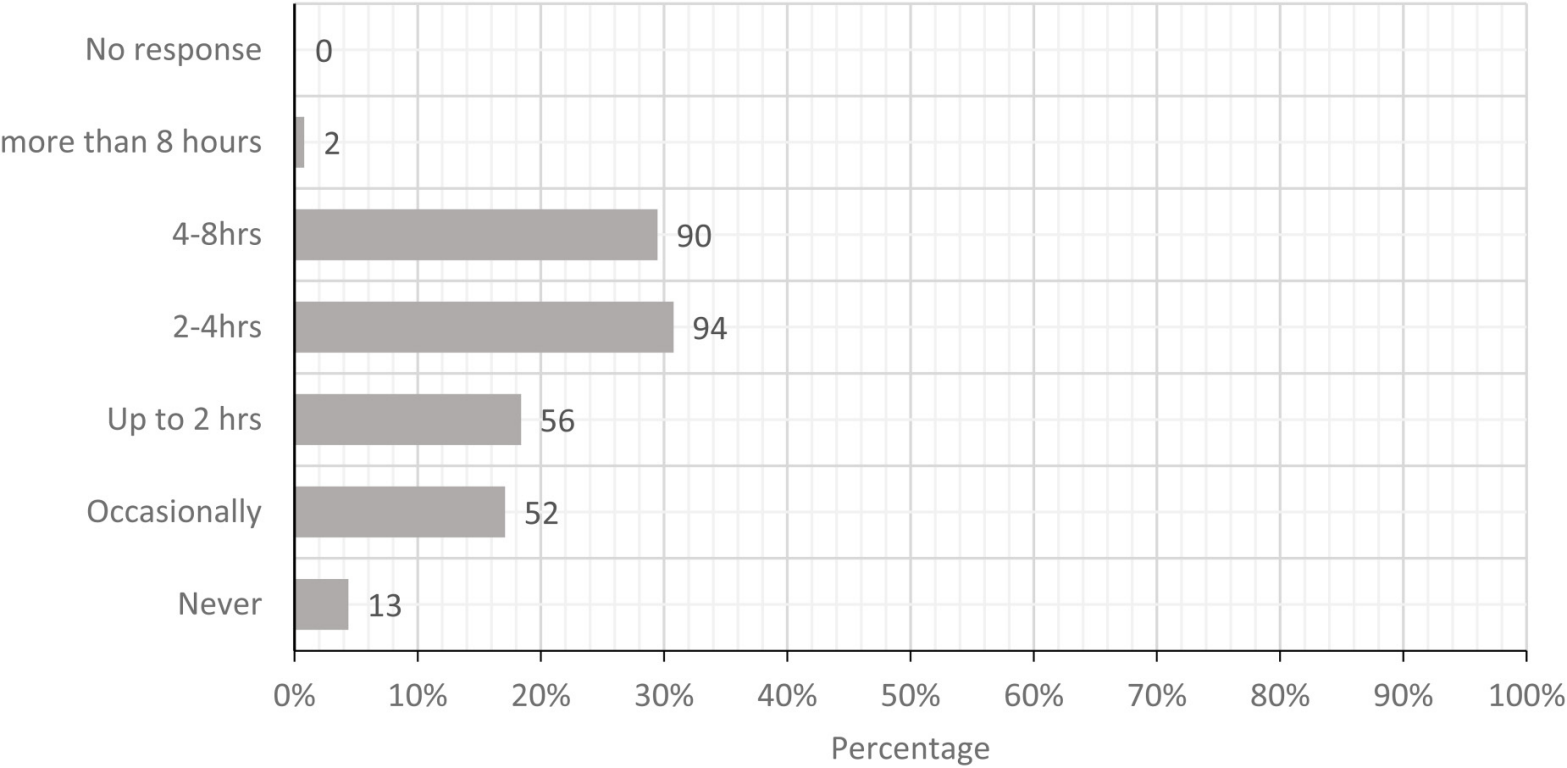
Today, how often did you stand with infrequent walking?

**Figure 3.**
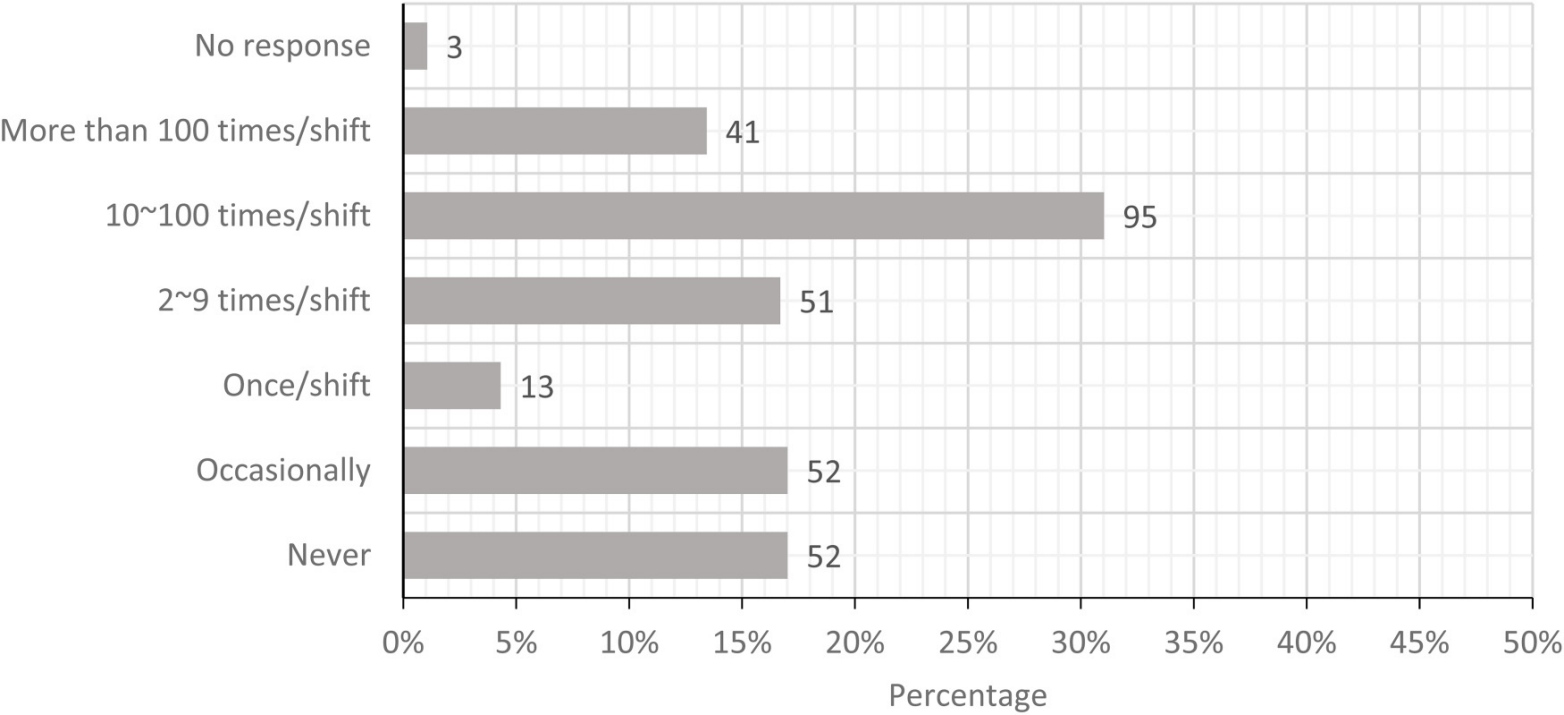
Today, how often did you carry loads more than a few steps (loads greater than 10 kg or 25 lbs.)?

**Figure 4.**
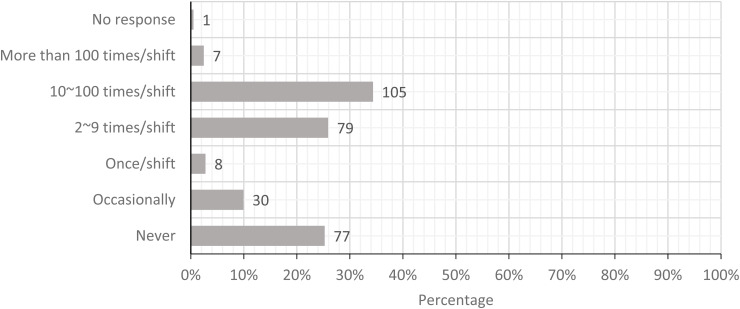
Today, how often did you push or pull loads more than a few steps: wheeling more than 100 kg (225 lbs) or dragging more than 35 kg (75 lbs)?

**Figure 5.**
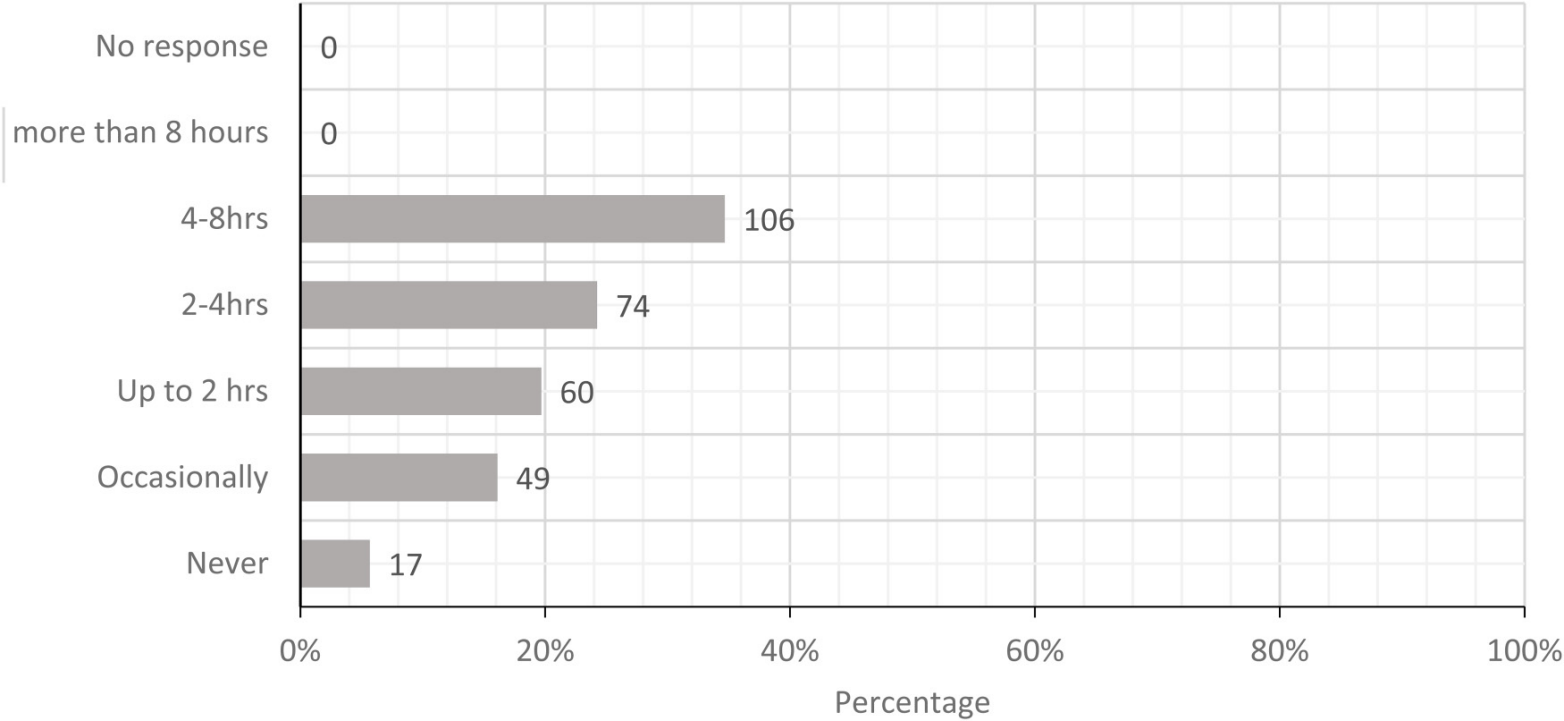
Today, how often did you perform repetitive movement of the whole arm more than twice per minute?

**Figure 6.**
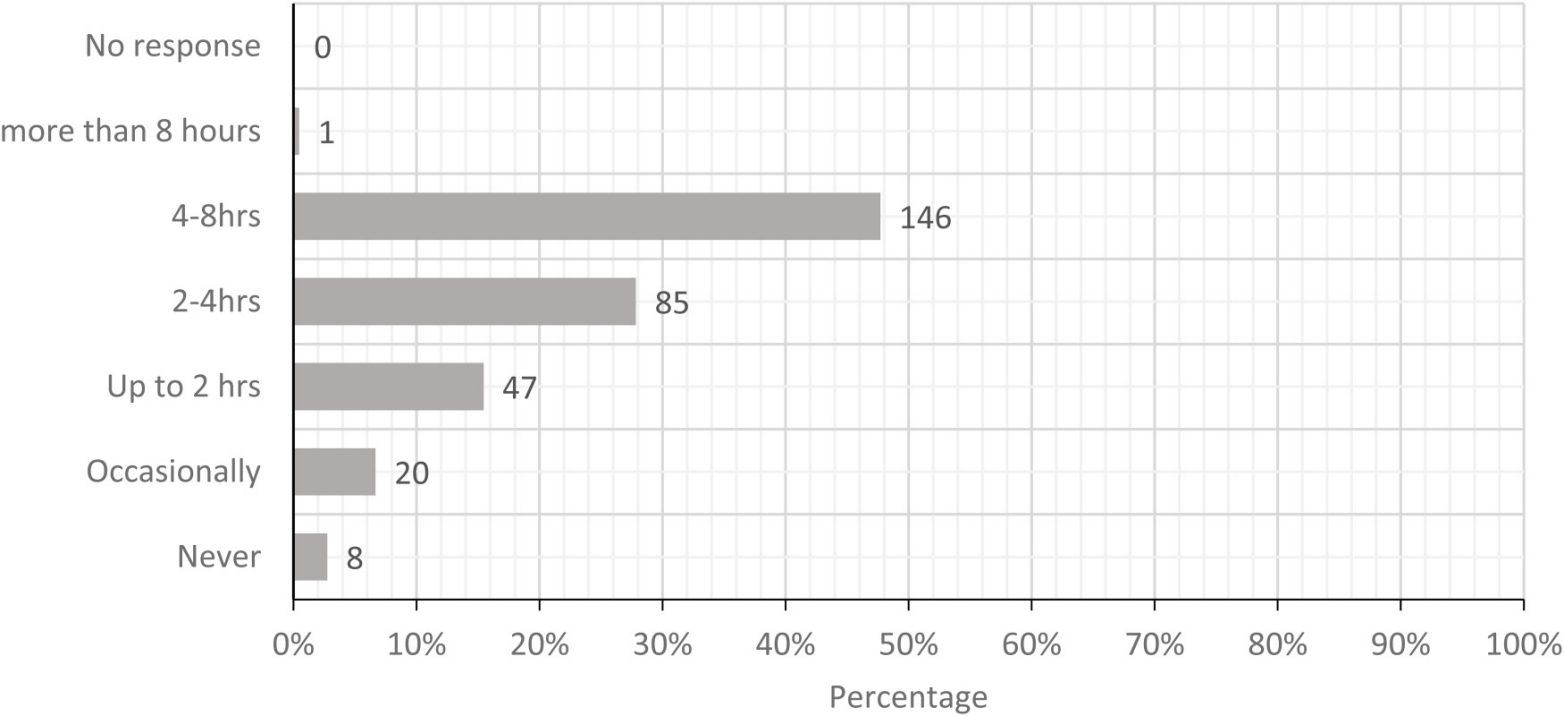
Today, how often did you move your hand, wrist, or forearm more than 10 times per minute not typing?

The five most prominent physical loads were standing with limited walking, carrying loads greater than 25 lbs, pushing or pulling loads greater than 225 lbs, performing whole arm movements more than 2 times/minute, and movement of hand and forearm more than 10 times/minute. Figures 2 to 6 show the workers’ estimates of their exposures. If the participants were not able to calculate the specific frequency, the PLS allowed participants to provide a descriptive estimate (never and occasionally). In summary, these workers categorized their exposure to physical loads in order of magnitude: awkward position, force, and repetitive movement.

Finally, the survey data indicated that the warehouse workers had fewer concerns than the retail workers. This was unexpected since it might have been assumed that they would have more exposure to ergonomic hazards. A possible explanation is that warehouses have the equipment necessary to handle larger volumes and packages of product. During the interviews, the participants reinforced the reports that the smaller workplaces cannot accommodate equipment like forklifts, and accordingly ergonomic solutions are limited. Not only did workers in small locations manually move more material around the store, but often had to unpack deliveries by hand (also known as de-stuffing the load).

### The Health and Safety Infrastructure

Because of the complicated health and safety infrastructure in this organization, the project team thought that the PHSC was the appropriate venue to initiate a discussion with the employer. It is important to remember that the PHSC has no authority either legislatively or corporately to initiate change independently, but it could ask for a program to be included on the corporate agenda. It was open to the project team members to report on the survey to their individual workplaces.

The university researcher presented a report to the PHSC which highlighted the survey results and identified the most common physical load exposures. This was the first opportunity for the project team to provide information to the management representative. In addition to the survey results, the researcher introduced a report from a similar workplace, which had developed a proactive ergonomic organizational program. That study established that a participatory ergonomic initiative had not only reduced claims but increased productivity and improved labor relations.^17^ At the same time, the university researcher also presented a proposal designed for another similar workplace to assist with cash stations.^18^ Because the employer member did not have corporate authority to take any action, consideration and decisions about the reports and possible programs were deferred until there were further decisions by the corporation.

### The Worker's Compensation System

In this jurisdiction, most injured workers are covered under a government-run system (WSIB) and there are 2 sub-branches in that scheme. Workers and employers covered under the first branch (Schedule 1) are protected from legal court cases by an insurance plan which is funded by employer premiums. This branch provides the employers with some protection from the full costs of injuries because of the principle of collective liability (all covered employers share the costs of all claims). The second branch, known as Schedule 2 (of which this organization is a part), covers large employers and most government workers. Schedule 2 employers are bound by the WSIB legislation and claims are managed by the WSIB, but the employer pays the full costs of all claims so there is no collective cost-sharing. The third smaller group is reserved for unscheduled employers and allows them to carry private insurance and does not limit legal claims. Because the employer is a Schedule 2 employer, any cost savings from a specific MSD prevention program could have an immediate positive impact on operating costs.^19^

### Workplace Locations and Organization

Another factor affecting the frequency of workplace injuries is the complex operation of this workplace, which is made up of stores and warehouses of various sizes province wide. Some of the retail outlets are in very old buildings, with layouts that make it impossible to mechanize shipping and receiving. In these workplaces, the staff manually load and unload all stock. Other stores are large and have physical space and mechanics for loading and unloading, called “pallet drop” stores. Some of the retail outlets employ only 2 staff, while others employ up to 100 staff. These operational characteristics limit the mechanization of tasks and preclude opportunities for job rotation, especially in small stores.

The workers also identified an unexpected issue. In the very large facilities, wrapped pallets of product arrive directly from suppliers. According to our participants, the suppliers employ short-term contract workers to wrap and prepare the products for delivery. These contract employees are not adequately trained and the wrapping process itself became an additional hazard. For example, if the wrapping is not applied correctly, it requires significant force to remove.

The participants also indicated that the cashier duties were the least desirable for several reasons. First, cashiers are restricted to 1 workstation with limited ability to move around. Cashiers are assigned to these duties for 3 h and are not usually provided with any seating. Second, cashiers are required to scan continuously resulting in excessive repetitive lower arm movement and lifting.

### The Organization of the Work Force

Another factor is the organization and composition of the workforce. There were four discrete worker groups included in the survey distribution.

1. Management in small stores who have the same responsibilities as bargaining unit members (eg, cash, stocking shelves, and loading and unloading shipments),
2. Full-time staff who are stationed at one location and have defined regular full-time hours, benefits, and pensions,
3. Casual regular part-time staff who can work at different locations, even on the same day. They can accrue sufficient hours to earn benefits and pension credits. Their hours vary and are assigned on a weekly basis by managers’ estimates of demand. Many of these workers have been deemed part-time for several years waiting for a full-time position to become available. These workers are union members and participate in H&S projects including this project,
4. Contract fixed-term staff. The fixed-term employees are hired for short contracts during busy seasons (eg, summer and Christmas seasons).

The casual staff are part-time regular staff who augment the needs of the employer on an ongoing basis. The terms of the current collective agreement have limited the casual staff to 70% of the workforce. Before 2017, casual staff may have composed up to 80% of the staff. A significant proportion of part-time employees have no benefit ties to the employer.

In its attempt to address the high number of MSDs, the employer initiated 2 programs, which are affected by staffing characteristics and are somewhat controversial. Because the current programs are in place, we have limited our considerations to what currently exists and have not addressed these controversies. One program deals with return-to-work following an injury. The second program deals with job rotation aimed at preventing injuries by sharing exposure to physical hazards. Although these programs could work in concert, they operate separately.

The return-to-work program encourages workers to return to work as quickly as possible with restricted duties. The controversy lies in the fact that workers are offered accommodated work immediately after injury, often before their individual restrictions, or even their medically sanctioned return-to-work dates, are known. According to worker reports, these injury accommodations mean that many staff cannot be rotated to certain tasks like unpacking loads or doing cash. In some workplaces, there are greater numbers of restricted employees than unrestricted employees and that increases the unrestricted workers’ exposure to physical loads.

The job rotation program is meant to be proactive. It aims to reduce exposure to physical loads for all workers. A daily schedule is posted in each workplace that is intended to help ensure that individuals spend a maximum of three consecutive hours on one task. However, according to union records, it was not unusual to have a higher number of restricted staff than unrestricted staff. Therefore, according to worker reports, the rotation schedule may not have been effective in limiting exposure to loads because rotation was not possible. In reality injury accommodations in the workplace (that are not shown or illustrated in the rotation schedule) override the rotation possibilities, making the schedule more of a plan than reality.

In addition to scheduling challenges, there are issues related to the status (permanent vs temporary, part-time vs full-time) of the workforce. In recent years, the staff has transitioned from majority permanent full-time to majority part-time. The union reports that the increase in part-time staff may be, in part, an initiative to reduce labor costs because of the reduction in the benefit costs. Having so many part-time workers on limited shifts is another reason that job rotation programs are difficult to organize and maintain. Irregular staffing practices reduce the likelihood of equitably sharing strenuous physical activities, which in turn also have the potential to adversely affect morale. Furthermore, although some part-time staff members have a long history with the employer, many are transient with no entitlement to benefits and accordingly have limited ties to the workplace. That lack of connection to the workplace, and in turn, the lack of commitment on the part of the employer to its transient staff has the potential to reduce the application and effectiveness of health and safety programs. Part-timers’ lack of attachment is not unusual and is well-documented in the literature reporting on Canadian and global workplaces. That research also confirms that health and safety programs can be less impactful because of transient workforces.^20–23^

## Discussion

Multidimensional knowledge concerning health and safety issues in this organization has been enhanced because of this research. The workforce has gained a marked increase in the technical knowledge about their exposure to physical loads. The collective activity by the union health and safety community enhanced their strategic knowledge. The value of this knowledge is explained by the HSO who stated:

When we first go in….well what do they (the academic researchers) need me for? But then that disappears, and you realize that you have something to bring. I felt like people (academics) respected the skills I had which weren't the same skills as they had.

The various forms of knowledge identified by the HSO and discussed in the introduction include—technical, strategic, and political knowledge. They lay along a continuum from the specific (technical) to procedural (strategic) and finally institutional (political). This case study documents the journey from the creation to the application of different forms of knowledge.

The project resulted in the development of technical knowledge, which produced a reliable survey that could be widely distributed. The labor team members provided practical knowledge about the organization to the academic team members. The academics gained insight into the duration of exposures, especially in a primarily part-time workforce. Accordingly, the survey instrument was modified to accommodate the nontraditional working schedules and allowed it to be used with ease by the participants. The academics provided knowledge about the foundations of a survey that could produce results, which would be persuasive to management and H&S policy experts. Together labor and academic researchers collaborated in a process that allowed for the viable distribution of the PLS. The academic members guided the processing and analysis of the data. The union members provided input that improved the tool's usability. As a result of this knowledge creation process, the newly created PLS is a validated tool that can be used systematically to identify hazards in this and other workplaces.

In addition to the development of the PLS, this project also identified hazards that needed attention, including at least 5 key physical loads to which a significant portion of the working population was exposed. The survey itself provided a framework for the monthly inspections conducted by the local H&S representatives. Site JHSC members can use the monthly survey results to contribute agenda items for discussion at JHSC meetings and make recommendations that legally require a formal response from the employer. The newly created technical knowledge and documentation of the workers’ exposure to physical loads has set the stage for a formal proactive collaborative ergonomic program, or at least the development of written recommendations to the employer.

From a strategic perspective, the creation of the PLS went beyond addressing the high number of MSD injuries to issues that affected LBED employees’ relationship with each other and the provincial union organization. Union members were afforded the opportunity to work together on issues that affected individual work sites and the entire organization. The union's support gave the PLS credibility for the workforce and involved members in a collective activity. The union financial resources also brought HSRs from across the province and union staff together to learn from each other about common hazards and solutions. Site-based HSRs and JHSCs (who operate independently) had a chance to communicate, compare notes, and work together on unifying health and safety initiatives. This is of particular value because the legislation does not include a mandate for HSRs and JHSCs at independent sites within a multi-site organization to interact or communicate about occupational health and safety. This broad-based collaboration was of particular benefit to the union because the employer does not acknowledge the PHSC as a legislated entity. While the PHSC provides a unified voice for LBED, it is a negotiated structure without the power that health and safety legislation has over individual work sites. Therefore, the PHSC has difficulty in gaining organization-wide improvements for hazards such as MSDs, where the employer believes their measures and procedures are already adequate.

The survey results also identified worker status (part-time or full-time) as a strategic consideration. Research has documented the negative impact of a contingent workforce on health and safety initiatives due to the limited time and opportunities that part-time and temporary workers have to engage in health and safety training and programs.^20–23^ Those research findings resonate in this case. The PLS responses showed a much lower response rate of part-time employees. It may be that part-timers are not interested, were missed in the distribution process, may think that their opinions do not count, or they may be excluded from participatory programs. Alternatively, part-time staff who received the email may be concerned that engaging in health and safety programs could negatively affect their relationship with management. That relationship is seen as a gateway to obtain preferred scheduled hours or other aspects of task assignments. Regardless of the reason, the limited input from part-time employees (who compose most of the workforce), throughout this process may also have a critical impact on health and safety initiatives. This work organization issue goes beyond the technical issues about exposures to specific physical loads and addresses systemic issues which impact the effectiveness of return-to-work and job rotation programs. Change that might result from the acquisition of this strategic knowledge is an issue that requires additional attention from both the employer and the union if MSD rates are going to be reduced.

Although the research has helped to create knowledge, there are barriers to change resulting from this new knowledge. A major stumbling block identified by Yazdani et al. and others is the negative impact of the absence of management's commitment to integrate health and safety into its business plans. Those researchers found that many individual ergonomic initiatives are implemented only on a short-term basis. Whereas business management frameworks that include ergonomic practices are continuously revisited, improved, and sustained.^3,12,13^ The other stumbling block is the need for sustained pressure from the union to promote an organization-wide MSD program. While we recognize that this union has multiple responsibilities, including the need to negotiate benefits and full-time wages, and the need to address other health and safety issues which include violence in this workplace, there is also a need to promote organizational MSD programs.

In this case, there may have been organizational change if the union and its LBED members had chosen to exert political knowledge to ensure that health and safety were not a sidebar issue but a critical component of any management system. However, based on experience, LBED did not believe they would have obtained the employer's support at the outset of the project. Therefore, LBED members made a strategic choice and undertook the project on their own with the hope they could bring results and evidence to the employer to encourage change. Unfortunately, employer participation was not forthcoming and organizational change has not yet come to fruition. Nonetheless, the worker representatives brought the issue forward themselves and did move ahead without the employer. There were changes associated with knowledge creation—albeit not the desired development of an organization-wide MSD prevention program.

We also began to understand how the development of technical knowledge can enhance existing knowledge and create new activism. Twenty years ago, in 1997, Krogh et al. challenged researchers (knowledge creators) to develop knowledge activists. Those authors saw knowledge activists as catalysts of knowledge creation, as connectors of knowledge creation efforts and merchants of foresight.^24^ This analysis is consistent with the more recent thoughts of others who have considered the application of knowledge. In their earlier work, Hall et al. recognized that knowledge activists were characterized by the wide-ranging base of their knowledge and the focus on the underlying causes of disabilities.^8^ Recently, Hall et al. advanced the discussion of political health and safety knowledge activism and were able to correlate the impact of this specific type of knowledge activism with its ability to create change. Specifically, they found that political knowledge resulted in change that went beyond the introduction of instrumental knowledge.^25^

We found that the road from the development of technical knowledge creation to political knowledge activism is not linear, but it exists. In the first instance, union representatives realized that they have more power when they help create documented technical knowledge and have access to that knowledge. When that knowledge is strategically used it has the potential to advance the development of proactive PE, a political goal. The fact that technical knowledge has not yet resulted in structural change, which would include collaborative proactive ergonomic programs, confirms Hall's conclusions that political (institutional) knowledge activism is critical. The union participants, in this case, now have a broader network of health and safety specialists and have gained technical and strategic knowledge during this process. The union in this workplace has used significant political knowledge to negotiate good working conditions and fair wages. There is now an opportunity to move health and safety issues to a political platform and bargain for systemic collaborative health and safety initiatives. This case has set the stage for further knowledge creation and further evidence-based workplace change.

## Limitations

There were some limitations in the data we collected. The survey should have included some demographic questions, such as gender, which could have provided a more detailed analysis. Also, the employment status of the workers (full or part-time) should be collected in the future because of its impact on the successful implementation of health and safety programs. Inclusion of gender and workers’ status in the survey questionnaire can be easily done in the future use of the survey. Additionally, we do not have a good estimate of the response rate. Originally, the decision to conduct the study was made by a small working group that did not represent all 500 work sites. Consequently, the decision to circulate the survey was not mandatory but left to the individual union representatives in each workplace and we did not calculate the total number of possible respondents. In the future, we would recommend that the number of possible respondents be recorded. That information along with the status of the workers should provide more insight into the nature of the hazards and possible solutions.

## Conclusion

Notwithstanding the limitations, it is important to recognize that the union and its membership were activists. They decided to proceed with a research initiative with or without management support to develop a technical knowledge base for future activities. It would have been easy to ask for management support and if that request was rejected to abandon the project. Instead, there was an independent decision to conduct research to improve individual workplaces and perhaps initiate more broad-based change. We have learned that the union's attention to health and safety issues and support for worker-suggested initiatives inspired members to become knowledge activists. At the instigation of a few knowledge activists, a greater number of workers became knowledge creators and may in turn become knowledge activists.

## Supplemental Material

sj-docx-1-new-10.1177_10482911221074680 - Supplemental material for The Path from Survey Development to Knowledge Activism: A Case Study of the Use of a Physical Loads Survey in a Retail Workplace Click here for additional data file. Supplemental material, sj-docx-1-new-10.1177_10482911221074680 for The Path from Survey Development to Knowledge Activism: A Case Study of the Use of a Physical Loads Survey in a Retail Workplace by Nicolette Carlan, Terri Szymanski, Jennifer Van Zetten, Margo Hilbrecht and Philip Bigelow in NEW SOLUTIONS: A Journal of Environmental and Occupational Health Policy
