# Supplementary material for: The Path from Survey Development to Knowledge Activism: A Case Study of the Use of a Physical Loads Survey in a Retail Workplace
Source: New Solut. 2022 Feb 4;32(1):65–76. doi: 10.1177/10482911221074680 (PMC9014667; doi:10.1177/10482911221074680)
Supplement: sj-docx-1-new-10.1177_10482911221074680 - Supplemental material for The Path from Survey Development to Knowledge Activism: A Case Study of the Use of a Physical Loads Survey in a Retail Workplace [file sj-docx-1-new-10.1177_10482911221074680.docx]

The Path from Survey Development to Knowledge Activism: A Case Study of the use of a Physical Loads Survey in a Retail Workplace

Nicolette Carlan, Terri Szymanski, Jennifer Van Zetten, Margo Hilbrecht, and Philip Bigelow

**APPENDIX A**


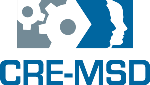
 Survey of Physical Loads at Work

Dear workers, union representatives and supervisors:

We are a research team, based at the University of Waterloo that is developing and validating a new kind of survey. The survey is designed to help Ontario workplaces and the Health and Safety System better understand physical loads at work.

We are asking for your name and/or email address so we can follow up in a couple of months to determine if you made any changes to your work as a result of the survey. If you do not feel comfortable providing us with that information, you can still access the survey and may find it beneficial.

The data is collected via REDCap software which is being widely used by clinical researchers for secure data collection and storage. The data will be stored on the REDCap server which is hosted at the University of Waterloo. The server is protected by UW's firewall and according to strict security and access control policy. Only authorized researchers with Research Ethics Approval can view data.

If you have any questions about this project or how to fill out this survey please contact: Niki Carlan, Project Coordinator at 519-739-3049 or ncarlan@uwaterloo.ca.

HOW TO FILL OUT THIS SURVEY

We would like you to estimate how often you do the activities described. In the survey to follow, we have included some pictures of tasks that could fit each question, however these are only examples.

The survey is designed to be completed by a worker. There is another version of this survey that can be used by joint health and safety committee members during routine audit activities. If you find an area where there is high exposure to physical loads you may want to alert the JHSC so they can do a formal hazard identification or risk assessment.

When completing the survey estimate the work being done today. We anticipate a good sample of participants so we should capture slow and busy days.

This survey was developed by adapting and modifying many of the questions in the Washington State Survey (Foley M, Silverstein B, Polissar N, and Neradilek B. (2009) Impact of implementing the Washington State ergonomics rule on employer reported risk factors and hazard reduction activity. American Journal of Industrial Medicine 52(1): 1-16.)

Ethics Statement

Participation in this study is voluntary. There are no known or anticipated risks to the company or to the workers as participants. You may decline to participate or you can withdraw at any time, and you can refuse to answer any question. Your withdrawal at any point will not have a negative impact on your relationship with our organizations or your company.

The researchers will keep the information you provide confidential. The name of your organization will not appear in any article, documentation or reports and will be referred to by number only in final documents. With your agreement, anonymous quotations may be used but attributed only to a general title (manager, worker health and safety representative) and in ways that ensure you cannot be identified. These assessments and notes will be maintained in a locked filing cabinet, in room LHI 3711, at the University of Waterloo for a period of four years (the period of time necessary for review and publication of research articles) and then destroyed.

| To help us assess the effectiveness of the tool, please provide us with your organization and your contact information for analysis and follow-up interviews. |
| --- |
| A. Please select where you are doing the survey: At Work  At Home |
| B. Can we contact you in a couple of months to determine if the survey Yes results have affected your workplace?  No |
| C. What is your position in your organization? |
| D. Please select the shift of the survey: Day  Afternoon  Night |
| E. How long is your shift today?   1. hour 2. hours 3. hours 4. hours 5. hours 6. hours 7. hours 8. hours 9. hours 10. hours |

| Section 1  Lifting / pushing / carrying / climbing |
| --- |
| Question 1: Today how often did you carry loads more than a few steps (loads greater than 10 kg or 25 lbs.)?  Examples: lifting crates from table to table more than a few feet apart, carrying construction material to site, moving furniture, moving carcasses.   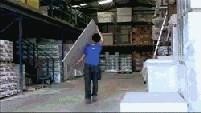 Never  Occasionally  Once/shift  2~9 times/shift  10~100 times/shift  More than 100 times/shift |
| Question 2: Today how often did you push or pull loads more than a few steps: wheeling more than 100kg (225lbs) or dragging more than 35kg (75lbs) ?  Examples: pushing trolleys, carts, and wheeled cages, material handling, drag or slide objects on the floor or table.   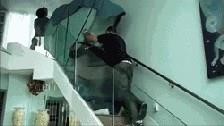 Never  Occasionally  Once/shift  2~9 times/shift  10~100 times/shift  More than 100 times/shift |
| Question 3: Today, how often did you lift or lower 23kg or 50 lbs., or more unassisted?  Examples: lifting cement bags, using a vacuIift to lift retail products, heavy boxes of paper, lifting heavy mattresses.    Never 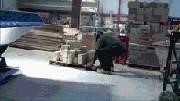 Occasionally  Once/shift  2~9 times/shift  10~100 times/shift  More than 100 times/shift |
| Question 4: Today, how often did you lift or lower 40kg (80lbs), or more unassisted?  Examples: jack leg drill in mining, lifting construction materials, unloading trucks, lifting piano (not people).  Never 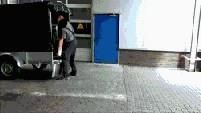 Occasionally  Once/shift  2~9 times/shift  10~100 times/shift  More than 100 times/shift |
| Question 5: Today, how often did you lift or reposition people unassisted?  Examples: lifting or transferring people into a wheelchair or toilet or bed, repositioning a person in bed.   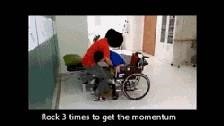 Never  Occasionally  Once/shift  2~9 times/shift  10~100 times/shift  More than 100 times/shift |
| Question 6: Today how often did you climb more than 8 steps on stairs or a ladder?   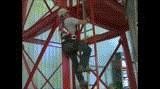 Never  Occasionally  Once/shift  2~9 times/shift  10~100 times/shift  More than 100 times/shift |

| Section 2  Loads related to awkward positions |
| --- |
| Question 7: Today how often did you bend forward with few pauses?  Examples: tying rebar on the ground, working on a low table, working with limited headroom, filling deli counters.  Never 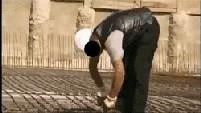 Occasionally  Up to 2 hrs  2~4 hrs  4~8 hrs  More than 8 hrs |
| Question 8: Today how often did you work with one or both hands above shoulder level with minimal loads in the hands with few pauses?  Examples: Painting overhead, installing wiring in the ceiling, sanding dry wall, garage mechanic working on the underside of car.   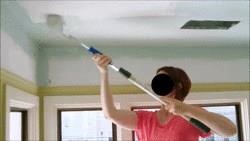 Never  Occasionally  Up to 2 hrs  2~4 hrs  4~8 hrs  More than 8 hrs |
| Question 9: Today how often did you lift or lower objects above the shoulders?  Examples: Installing overhead ductwork, loading boxes on or off high shelf.   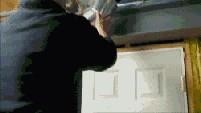 Never  Occasionally  Up to 2 hrs  2~4 hrs  4~8 hrs  More than 8 hrs |
| Question 10: Today, how often did you work with your neck in an awkward position with few pauses?  Examples: viewing a monitor placed off to the side, working cash looking up when painting a ceiling, holding a phone to your ear with your shoulder.  Never 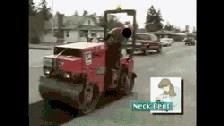 Occasionally  Up to 2 hrs  2~4 hrs  4~8 hrs  More than 8 hrs |
| Question 11: Today, how often did you hold a fixed position of the upper body with few pauses?  Examples: microscopic work, soldering small parts, packaging, inserting electronic components, sewing   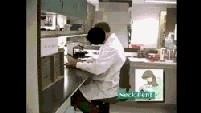 Never  Occasionally  Up to 2 hrs  2~4 hrs  4~8 hrs  More than 8 hrs |
| Question 12: Today how often did you kneel or squat?  Examples: Laying tile flooring, carpeting, landscaping, scrubbing floors  Never 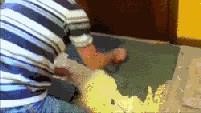 Occasionally  Up to 2 hrs  2~4 hrs  4~8 hrs  More than 8 hrs |

| Section 3  Loads related to repetitive hand and arm use |
| --- |
| Question 13: Today, how often did you perform repetitive movement of whole arm more than twice per minute?  Examples: Packing boxes, planting trees, feeding parts into a machine, mopping floors, cleaning wall with a cloth, wiping off tables, using an axe, opening and closing machine, weighing.   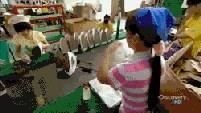 Never  Occasionally  Up to 2 hrs  2~4 hrs  4~8 hrs  More than 8 hrs |
| Question 14: Today, how often did you move your hand, wrist, or forearm?  Examples: Scanning groceries, chopping vegetables, packing small object, dealing cards, using a manual screwdriver  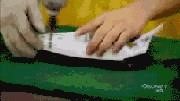.  Never  Occasionally  Up to 2 hrs  2~4 hrs  4~8 hrs  More than 8 hrs |
| Question 15: Today how often did you use a key board or mouse intensively?  Examples: Data entry, word processing, computer graphics.   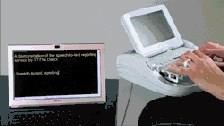 Never  Occasionally  Up to 2 hrs  2~4 hrs  4~8 hrs  More than 8 hrs |

| Section 4  Loads related to the use of force |
| --- |
| Question 16: Today how often did you pinch grip small objects between thumb and finger continuously or with few pauses?  Examples: Inserting small parts, using a dental tool, pipefitting, pinch  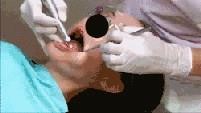.  Never  Occasionally  Up to 2 hrs  2~4 hrs  4~8 hrs  More than 8 hrs |
| Question 17: Today how often did you use your whole hand to grasp objects continually or squeeze objects repeatedly?  Examples: Using caulking gun, using pliers to tie rebar, pruning, holding knife in meatpacking, use hand tool, carry suitcase or bucket by handle.  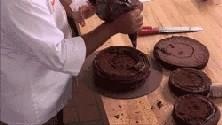.  Never  Occasionally  Up to 2 hrs  2~4 hrs  4~8 hrs  More than 8 hrs |
| Question 18: Today how often did you use your hand as a hammer to pound objects?  Examples: Breaking down boxes, banging in parts.   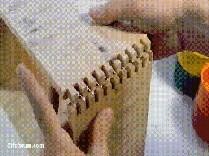 Never  Occasionally  Up to 2 hrs  2~4 hrs  4~8 hrs  More than 8 hrs |
| Question19: Today how often did you grasp objects while wearing heavy gloves or gloves that get in the way?  Examples: Handling cold product in freezer, meat cutting, maintaining live power lines.    Never  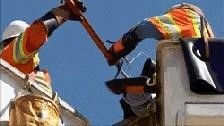. Occasionally  Up to 2 hrs    2~4 hrs  4~8 hrs  More than 8 hrs |
| Question 20: Today how often did you use your knees as a hammer more than once/minute?  Examples: knee kicker for carpet installation, loading skids, breaking boxes, making beds.  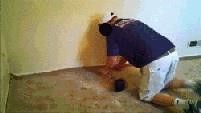.  Never  Occasionally  Up to 2 hrs  2~4 hrs  4~8 hrs  More than 8 hrs |

| Section 5  Loads related standing, sitting and vibration |
| --- |
| Question 21: Today how often did you stand with infrequent walking?  Examples: cashier, bank teller, machine operator, retail.  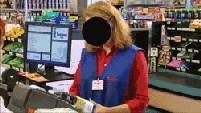.  Never  Occasionally  Up to 2 hrs  2~4 hrs  4~8 hrs  More than 8 hrs |
| Question 22: Today how often did you sit not in vehicles?  Examples: monitoring video screens, computer work, small parts assembly.   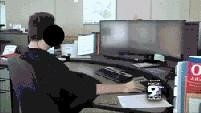 Never  Occasionally  Up to 2 hrs  2~4 hrs  4~8 hrs  More than 8 hrs |
| Question 23: Today how often did you sit in or drive on-road vehicles only?  Examples: Ride in or drive cars, buses, transport truck, pickup.  Never 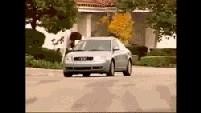 Occasionally  Up to 2 hrs  2~4 hrs  4~8 hrs  More than 8 hrs |
| Question 24: Today how often did you sit or stand on vibrating surfaces machines or off-road vehicles?  Examples: Forklifts, off road vehicles, construction vehicles, logging trucks, earth graders, forestry machinery, vibrating platforms.   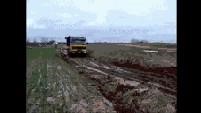 Never  Occasionally  Up to 2 hrs 2~4 hrs  4~8 hrs  More than 8 hrs |
| Question 25: Today how often did you work in the cold?  Examples: Outside workers, cold store or refrigeration workers.   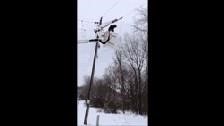 Never  Occasionally  Up to 2 hrs  2~4 hrs  4~8 hrs  More than 8 hrs |

| Section 6  Loads related to hand arm vibration |
| --- |
| Question 26: Today, how often did you use or grasp high** vibration tools or objects? moderate.  Example: use a hand wood sander, floor polisher, zamboni or floor cleaner, power drill, wizard knife.  Never 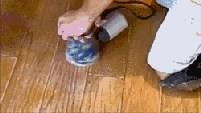 Occasionally  Up to 2 hrs  2~4 hrs  4~8 hrs  More than 8 hrs |
| Question 27: Today, how often did you use or grasp moderate** vibration tools or objects?  **Moderate is less than 8 hour energy equivalent frequency weighted acceleration value of 2.5m/s2.”  Example: use a chain saw, pneumatic chipper, hammer drill (or rotary hammer), grinder.   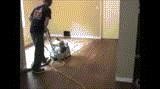 Never  Occasionally  Up to 2 hrs  2~4 hrs  4~8 hrs  More than 8 hrs |

Thank you for participation and finishing the survey!
